# Metals and Alloys

**Published:** 1863-05

**Authors:** B. Wood


### Original Essays and Communications

### METALS AND ALLOYS.

BY DR. B. WOOD.

(Continued from page 154, Vol. 17.)

### ZINC.

Hardness 7. Flex. 3. Mall. 3. Fusibility 7.

Zinc, frequently called "Spelter" in commerce, is a hard, strong metal, of a bluish-white color, semi-malleable, somewhat flexible, and melts at a dull red heat; or, according to different authors, at from 666° to 773 Fahr. Its fracture is crystaline and has a mild, bluish-white lustre. It has much greater tenacity than bismuth or antimony, and even when cast can be hammered down to thin plates, which, however, break at the margins. At about 300° Fahr. it is highly malleable, and may be rolled into thin sheets. When perfectly pure it is said it may be reduced to thin leaves at ordinary temperatures, but zinc of commerce requires for this a temperature above 200°. At about 400° it becomes brittle, and at a still higher temperature, near the point of congelation, may be triturated to powder.

In tables prepared by Calvert and Johnson, 1858, zinc is placed in the scale of hardness between silver and gold. In this scale cast-iron stands the highest, and is represented by 1000; copper, 301; silver, 208; zinc, 183 gold, (pure)

167. Brande, (according to Webster,) places it below gold, and between antimony and gold. In this scale bismuth stands above gold! Dr. Austen, (see experiments, American Journal Dental Science,) makes it harder than copper, and says, "Antimony and bismuth both rank lower in the scale of hardness than zinc." Bismuth is certainly much softer than zinc, according to any of the methods adopted for testing their quality. But antimony, according to one method, would appear softer; to another, harder. It scratches zinc, but on the other hand, will not resist so heavy a blow or weight. When triangular pieces of the two metals are brought together at the edges, and strongly compressed, in the manner described in the commencement of these papers, the antimony slightly indents the zinc piece, but on continuing the pressure, crushes under it *en masse*, though it has sufficient strength to cut the softer metals without suffering fracture by them. It is composed of harder particles, but less compactly cemented than zinc.

Zinc, it is said, "shrinks but little as it cools," (Piggott,) but the shrinkage, as well as warpage, under certain conditions, would appear to be considerable. The following will illustrate the trouble it may occasion in making casts for striking up plates for artificial teeth. A zinc bar 10 inches long by 3 wide and ½ thick, cast in sand, did not appear to have warped any, but shrank in its length three sixteenths of an inch. Casting over this a corresponding bar, so as to overlap the ends of the first, and thereby prevent sliding, it resulted in a perfect fit of the two halves, but a warpage of half an inch, reckoning from the extreme ends, the two pieces describing the segment of a circle. The force of contraction of the part last cast was here sufficient to bend the other as in a vice. Doubtless, a similar *springing* of *dies* when taking dental casts has frequently been the source of the trouble attributed to the "springing of plates."

Zinc readily oxidizes at a dull red heat; at full redness it burns with a dazzling white flame, and the flocculent oxide formed is driven off by the force of combustion, like a white cloud. Melted under a blow-pipe, this effect has a very picturesque appearance, the fumes of zinc rising in a white, gauzy column, like a spider's film, and standing in the form of a pyramid, its base attached to the coal, and its airy apex floating upward.

Zinc in dry air preserves its color, but loses its lustre; in damp air it oxidizes on the surface and turns gray. It dissolves in most of the acids with disengagement of hydrogen. It is also readily acted upon by alkalies, either in the metallic or oxidized form.

Most metals are precipitated in the metallic state by zinc from their solutions; it precipitates nearly every metal, either as such, or as an oxide.

Zinc is extracted from the native carbonate, (calamine,) or sulphuret, (blend,) generally from the former, from which it is reduced with charcoal, and obtained by distillation. The zinc in market is generally very impure, containing copper, arsenic, lead, iron, sulphur, manganese, &c. That produced in certain localities contains cadmium in such proportion as to afford a source of supply of the latter metal. The volatile portion of the impurities distils over with the zinc so that it is impossible to obtain it entirely free from them in the dry way, and resort must be had to solution in acids. To get rid of foreign matters sufficiently for ordinary uses, stir into the melted zinc a mixture of fat and sulphur, and skim off the sulphurets. "Zinc may be obtained nearly approaching to purity as follows: Commercial zinc is dissolved in dilute sulphuric acid, and in the solution a strip of the same metal is placed, remaining some hours. The liquor is now filtered and decomposed by carbonate of potash, and the precipitate, well washed, heated with powdered charcoal in a wide mouthed earthen or iron retort. The zinc, volatizing at a white heat, is distilled over in a vessel of water placed to receive it." (*Phillips.*)

The alloys of zinc are various, of which those with copper, forming the different varieties of brass, are the most important. Fine brass affords an example of an *alloy* which resists oxidation better than either of its constituents. This form of alloy has received such thorough attention from practical writers, that it would be superfluous, if not presumptous, for me to attempt to add any thing here.. It alloys with most other metals, but for some has a very weak affinity, or even repulsion, as lead, bismuth and nickle. With tin it forms very hard and strong alloys, which are malleable, and, to some extent, flexible, according to the preponderance of tin. These compounds undergo considerable waste in melting, in the form of drops, owing to semi-oxidation under heat. It does not combine so well with antimony, undergoing, under ordinary management, much loss by oxidation: the compound is brittle, though quite strong, and very hard. With cadmium when zinc is in excess the alloy is harder than zinc, resistant under the hammer, somewhat brittle, but possesses considerable malleability. Its behavior with lead and bismuth was noticed when speaking of those metals. It renders the less fusile metal brittle, except copper, but with this, in certain proportions, produces very strong and malleable alloys. In small proportion with silver or gold, it confers fusibility without so impairing their tenacity but that the compounds may be used as solders for these metals; but the addition of copper improves the result for this purpose. It is said to combine freely with aluminum, lowering its melting point, and forming a good solder for it. It has a feeble affinity for platinum, but a portion unites with it, resulting in a dark alloy that crushes to black powder under the hammer: copper added renders it somewhat malleable—color, silver-white and brilliant. A small proportion of zinc and antimony with tin constitutes an excellent die metal for swaging plates. The *alloy* of tin and zinc combines homogeneously with bismuth. With tin and cadmium it makes a very hard and strong alloy.

The uses of zinc in the arts in the form of sheet zinc, in the preparation of brasses, &c., as well as for the construction of galvanic batteries, also in the form of oxide for making paints, etc., is too well known to need mention here. It is employed as coin in China; and, it is said, of the utmost purity. In fireworks in filing, or particles, it produces beautiful spangles and stars of a fine blue color. Medicinally, it is never employed in the metallic state, but as an oxide, chloride, acetate, carbonate, sulphate, &c., the chloride, oxide and sulphate internally as well as externally, the acetate and carbonate externally only. Internally its general effect is tonic, astringent, and, in large doses, emetic. Externally it is applied to ulcers as an astringent or discutient. The chloride is employed to allay the irritability of sensitive dentine with some, but not with very marked advantage.

Metallic zinc does not appear to have been used in operative dentistry, (unless in the form of amalgam,) but the oxide of zinc, combined with fluid into a paste, constituting the osteoplasty as artificial, or "bone filling," brought to the notice of the profession a few years ago, is now used quite extensively for stopping carious teeth. The fluid generally used is chloride of zinc, (obtained by solution of zinc in muriatic acid,) although the chloride of several other metals answers the same purpose, and hydrochloric acid, (chloride of hydrogen,) in the hydrated form, as the muriatic acid of commerce, may be substituted for any of them with the same effect, and appears indeed to be the true agent in producing the result, whether applied directly in its simple state or indirectly as holding a metal in solution. The paste hardens in a few moments to a solid, similar to plaster of paris mixed with water, and is insoluable in water. It is, however, readily dissolved in acids, however feeble or diluted, and alkalies, as well as other reagents. That it is not disintegrated in the mouth in a few days proves that the mouth, instead of being an alembic wherein all soluble substances, particularly metals, and, as some contend, even gold itself, are liable to corrosion, and to transmutation into pernicious oxides, is on the contrary, when in a normal condition, (a condition, however, too seldom found,) rather a conservator. It demonstrates what no chemical analysis, or examinations with test paper have satisfactorily determined, or, perhaps, are competent to determine, that the normal fluids, instead of being acid or alkaline, or corrosive in their action, are as nearly neutral as it is possible to be, and as inert as water. When otherwise it is due to disease, or to the decomposition of foreign matter lodged about the teeth, which decomposition the buccal fluids naturally serve to check, being eliminated in quantity and quality suited to this effect. But for this, the teeth of every person—of every living being, granivoræ, carnivora, or herbivora, would be decayed. The best *tooth wash* in the world is healthy *saliva*, excited to secretion by friction, or by saline, saccharine or other natural stimulent.

It is chiefly in the laboratory that zinc becomes of importance to the dentist, where its hardness and strength render it of great value for dies in conjunction with moulds made of a softer metal. It has the disadvantages of shrinking considerably, and requiring an inconveniently high heat for its fusion; but it forms alloys with tin and antimony, which obviate these defects.

We here bring this series of papers to a close. I proposed at the start to pursue the subject from time to time, as occasion offered. I now take a respite, regretting, however, that circumstances have prevented the bestowment of more care in respect to style and arrangement. It only remains to arrange in tabular form the specific formulæ of alloys, the general characters of which have been described, which will be presented hereafter in a separate paper.

ALBANY, N. Y.

